# Ultrasound-Guided Retroperitoneoscopic Lumbar Sympathetic Chain Selective Clamping with Indocyanine Green-Assisted Access: A Technical Note

**DOI:** 10.3390/jcm15166203

**Published:** 2026-08-11

**Authors:** Vincenzo Schiavone, Gennaro Giulio Melone, Pierpaolo Castagliuolo, Giuseppe Sgarlato, Alessandro Costa, Rossana Alloni, Gianluca Mascianà, Enrico Davoli

**Affiliations:** 1 Department of Advanced Biomedical Sciences, University of Naples “Federico II”, Via Sergio Pansini, 5, 80131 Naples, Italy; vincenzo.schiavone@unina.it; 2 Department of General Surgery Resident, Università Campus Bio-Medico, Via Alvaro del Portillo 200, 00128 Rome, Italy; p.castagliuolo@unicampus.it; 3 Operative Research Unit of Anesthesia and Intensive Care, Fondazione Policlinico Universitario Campus Bio-Medico, Via Alvaro del Portillo 200, 00128 Rome, Italy; g.sgarlato@policlinicocampus.it; 4 UniCamillus School of Medicine, Saint Camillus International University of Health and Medical Sciences, Via di Sant’Alessandro, 8, 00131 Rome, Italy; u.005553@students.unicamillus.org; 5 Sarcoma Surgery, Fondazione Policlinico Universitario Campus Bio-Medico, Via Alvaro del Portillo 200, 00128 Rome, Italy; r.alloni@policlinicocampus.it; 6 Fondazione Policlinico Universitario Campus Bio-Medico, Via Alvaro del Portillo 200, 00128 Rome, Italy; g.masciana@policlinicocampus.it (G.M.); e.davoli@policlinicocampus.it (E.D.)

**Keywords:** hyperhidrosis, plantar hyperhidrosis, selective clamping

## Abstract

**Background:** Primary plantar hyperhidrosis is a disabling condition characterized by excessive plantar sweating and impaired quality of life. Lumbar sympathectomy is reserved for patients with symptoms refractory to conservative treatment, but minimally invasive techniques remain technically heterogeneous. This study describes a modified retroperitoneoscopic lumbar sympathectomy and reports its preliminary perioperative outcomes. **Methods:** A single-center case series included consecutive patients who underwent modified retroperitoneoscopic lumbar sympathectomy for primary plantar hyperhidrosis between June 2019 and May 2024. The technique combined ultrasound-guided retroperitoneal access, indocyanine green (ICG)-assisted creation of the retroperitoneal working space, and temporary selective clamping of the lumbar sympathetic trunk with an intraoperative assessment of plantar temperature and perfusion before definitive clipping. **Results:** Forty-five patients underwent 81 procedures, including 35 bilateral procedures (70 sides), 10 unilateral procedures, and one unilateral reoperation for recurrent symptoms. All patients resumed oral intake and ambulation on the day of surgery and were discharged within 24 h according to institutional protocol. No intraoperative or postoperative complications were observed and resolution of plantar sweating in subsequent follow-ups was achieved. **Conclusions:** Modified retroperitoneoscopic lumbar Sympathetic Chain Selective Clamping appears to be a feasible and safe technique for the surgical treatment of primary plantar hyperhidrosis. These technical refinements may facilitate retroperitoneal access and intraoperative confirmation of the target sympathetic level. Larger comparative studies with standardized outcomes and longer follow-up are needed to confirm the durability and clinical value of this approach.

## 1. Introduction

Plantar hyperhidrosis is a chronic disorder characterized by excessive sweating localized to the soles of the feet, often occurring under normal thermal conditions and disproportionate to physiologic needs. It is frequently associated with palmar hyperhidrosis and may have a marked negative impact on quality of life, leading to social discomfort, reduced self-esteem, and functional limitations in daily activities [1]. In addition to its psychosocial burden, persistent moisture of the plantar surface may promote bacterial and fungal overgrowth, thereby predisposing patients to bromhidrosis and a variety of dermatologic complications, including tinea pedis, intertrigo, dyshidrosis, contact dermatitis, and secondary infections [2]. Several non-surgical treatment options are available for plantar hyperhidrosis, including topical agents, systemic anticholinergic medications, iontophoresis, and botulinum toxin injections. However, symptom control is often incomplete or temporary, and surgical sympathectomy remains a valuable option in carefully selected patients with disease refractory to conservative management [1]. Historically, treatment strategies have included open lumbar sympathectomy, percutaneous chemical or radiofrequency techniques, and minimally invasive endoscopic approaches [1]. Among surgical options, the retroperitoneoscopic approach has progressively gained interest because it combines the advantages of minimally invasive surgery with direct access to the lumbar sympathetic chain, potentially reducing postoperative morbidity and facilitating recovery [3]. Retroperitoneoscopy was first described in urologic surgery in 1995 [4], and shortly thereafter totally extraperitoneal laparoscopic lumbar sympathectomy was reported as a feasible approach [5]. Since then, endoscopic lumbar sympathectomy has been increasingly adopted for the treatment of plantar hyperhidrosis, with favorable reported outcomes in appropriately selected patients [6]. Despite these advances, minimally invasive lumbar sympathectomy remains less standardized than thoracoscopic sympathectomy, particularly with respect to retroperitoneal access, anatomical orientation during dissection, and intraoperative confirmation of the target sympathetic level. Variability in these technical steps may reduce procedural reproducibility and limit the broader adoption of the technique. Our approach does not substantially alter the definitive clip placement compared with previously described methods. Instead, it introduces technical refinements that improve anatomical orientation and facilitate a less invasive retroperitoneal approach. Specifically, the present study describes a modified retroperitoneoscopic technique for lumbar sympathectomy based on ultrasound-guided retroperitoneal access, indocyanine green-assisted creation of the retroperitoneal working space, and temporary selective clamping of the lumbar sympathetic trunk before definitive clipping. We report the preliminary perioperative outcomes from our single-center experience.

## 2. Materials and Methods

### 2.1. Study Design and Patients

This single-center retrospective case series included consecutive patients who underwent lumbar sympathectomy for primary plantar hyperhidrosis at Policlinico Universitario Campus Bio-Medico, Rome, Italy. All procedures were performed by the same surgical team using a standardized and reproducible operative technique.

Patients were eligible for surgery if they had a diagnosis of primary plantar hyperhidrosis, established based on clinical history and physical examination, with symptoms significantly affecting daily activities and quality of life despite previous conservative treatment. Conservative management included topical antiperspirants, botulinum toxin injections, or systemic medical therapy, according to individual clinical indications. Surgery was offered only to patients with persistent, refractory disease, in accordance with current surgical indications for lumbar sympathectomy [1,6]. Patients with secondary hyperhidrosis or incomplete clinical records were excluded from the analysis. A total of 45 patients were included (29 males and 16 females), with a mean age of 35 years (range: 19–62 years). Overall, 81 lumbar sympathectomies were performed, including 35 bilateral procedures (70 sides), 10 unilateral procedures, and one unilateral reoperation for recurrent symptoms. Clinical data were retrospectively collected from medical records and included demographic characteristics, operative details, perioperative outcomes, postoperative complications, symptom resolution and recurrence. Written informed consent was obtained from all patients prior to surgery, including consent for the surgical procedure and, where applicable, for the use of anonymized clinical data and images for scientific publication, in accordance with institutional policies.

### 2.2. Surgical Technique

**Figure 1:**
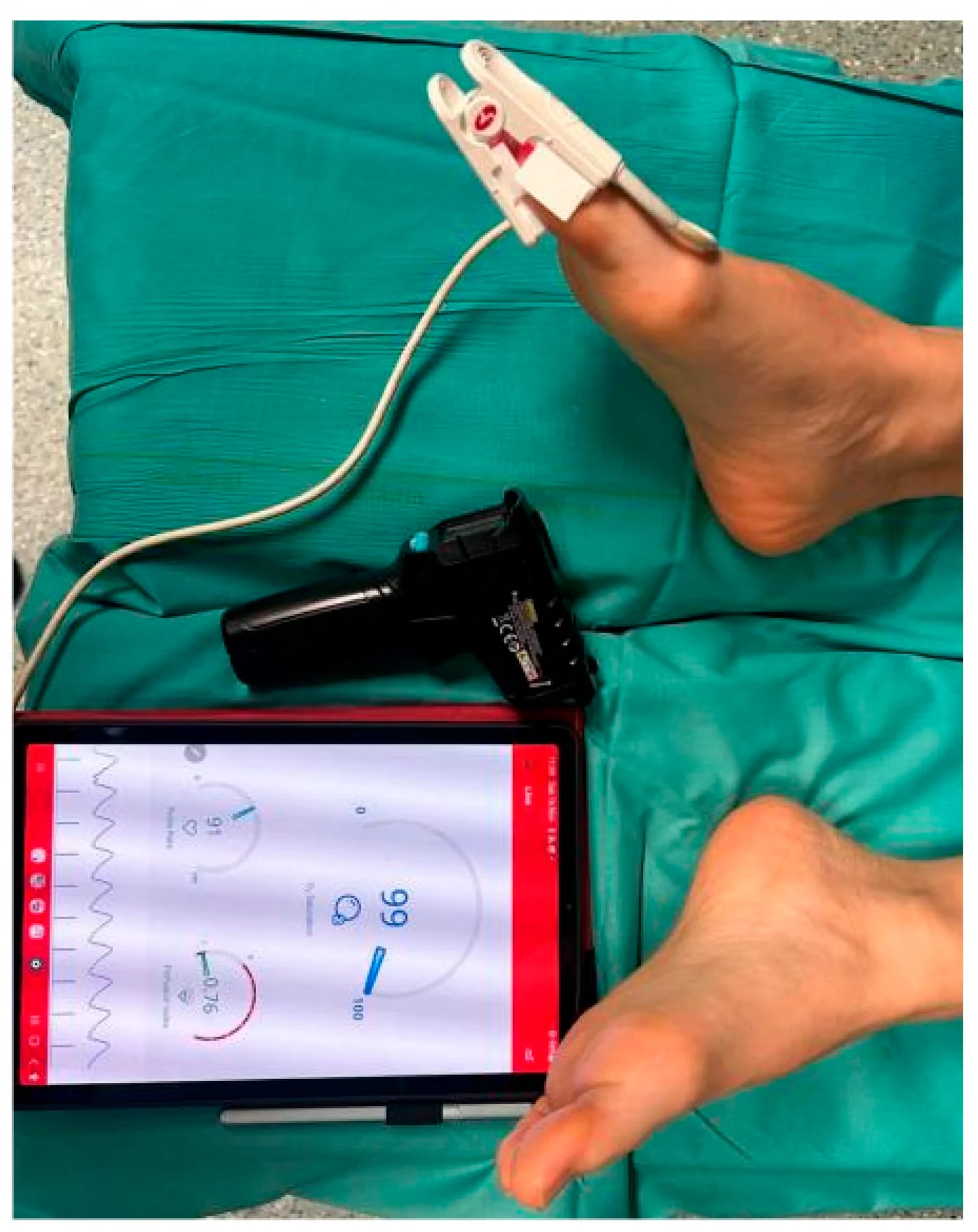
Application of a pulse oximeter to the big toe on the operated side, connected to a device capable of reading—among other things—perfusion oscillations.

**Figure 2:**
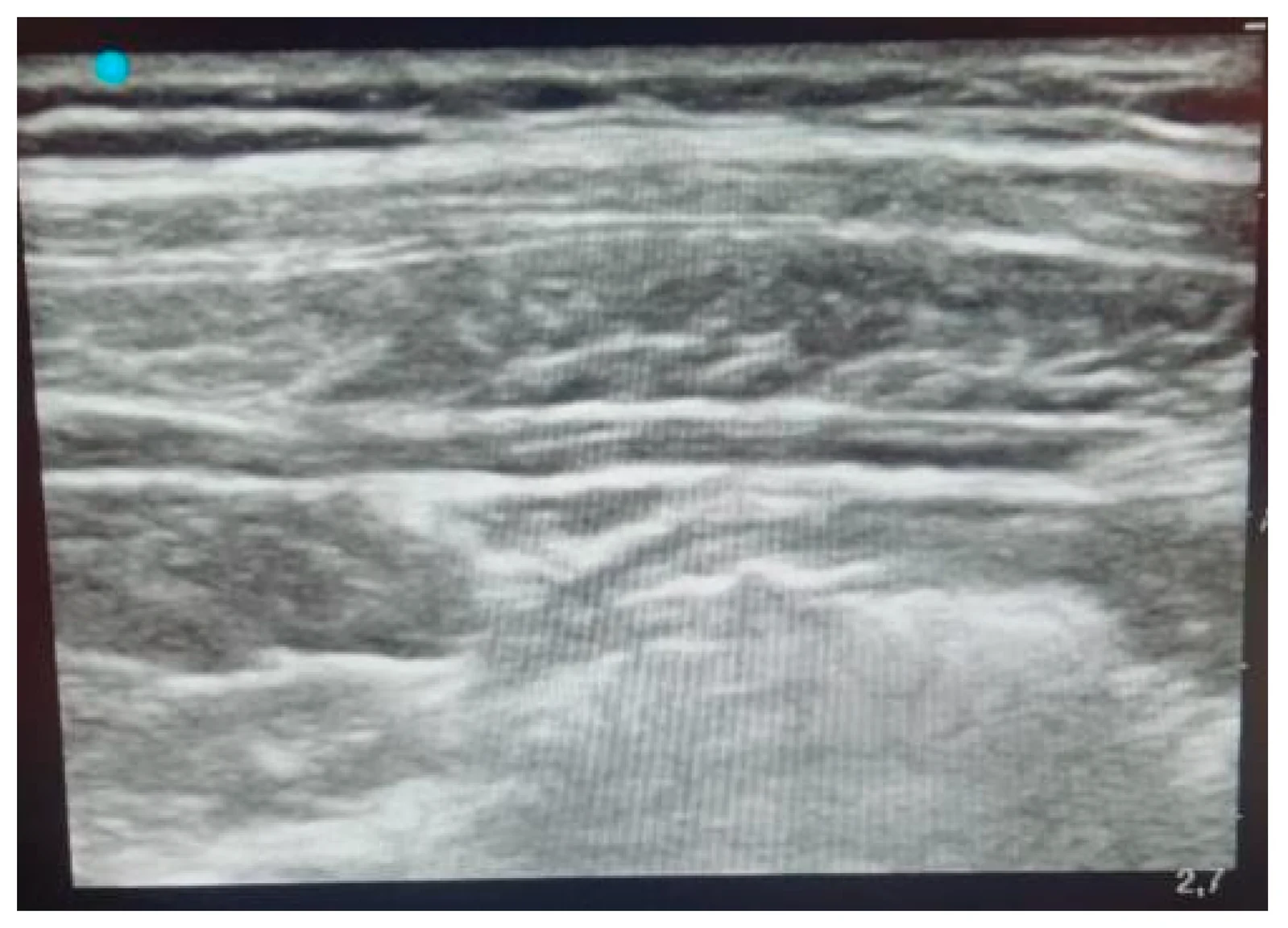
Ultrasound-guided creation of retroperitoneal space.

**Figure 3:**
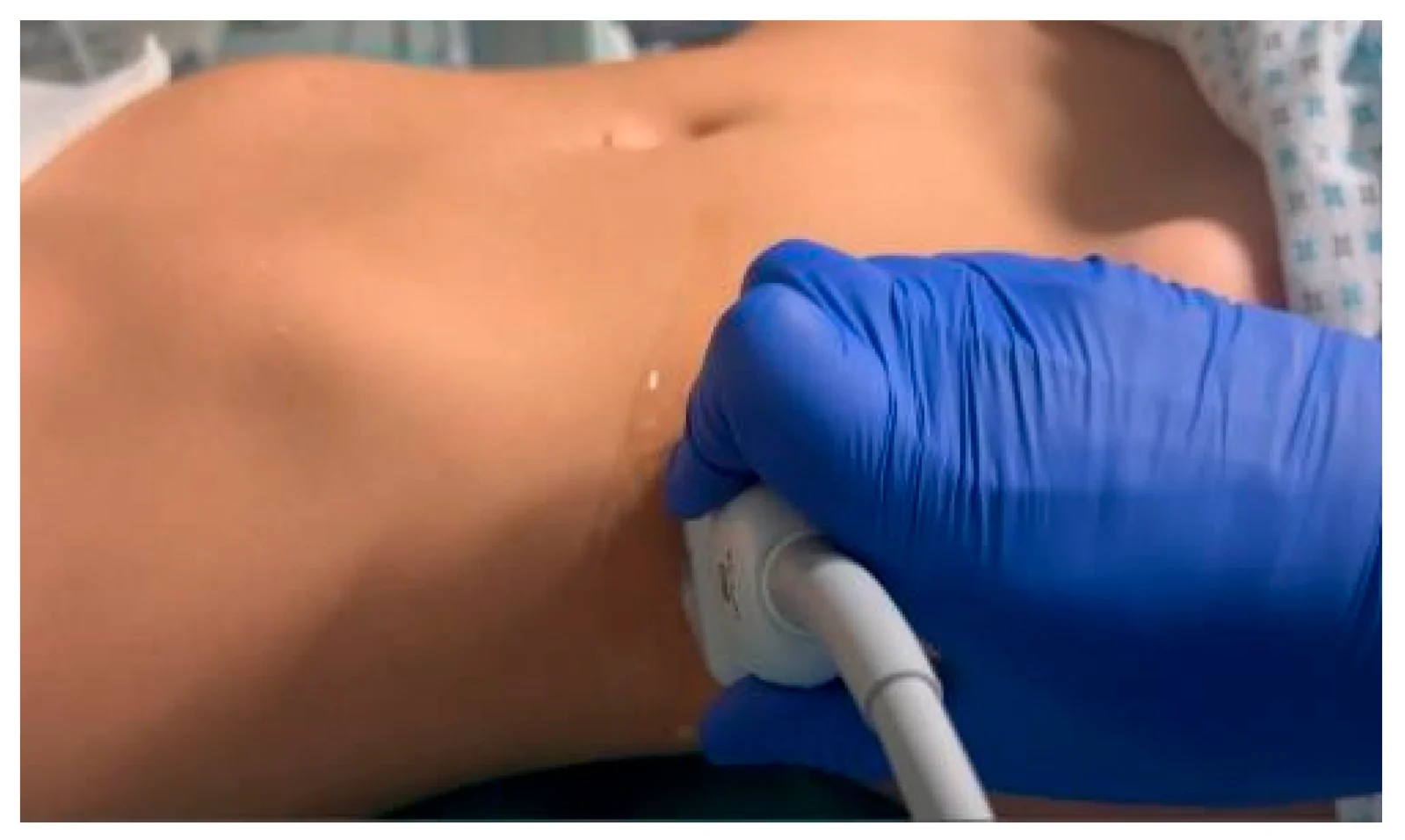
Application of the ultrasound probe.

**Figure 4:**
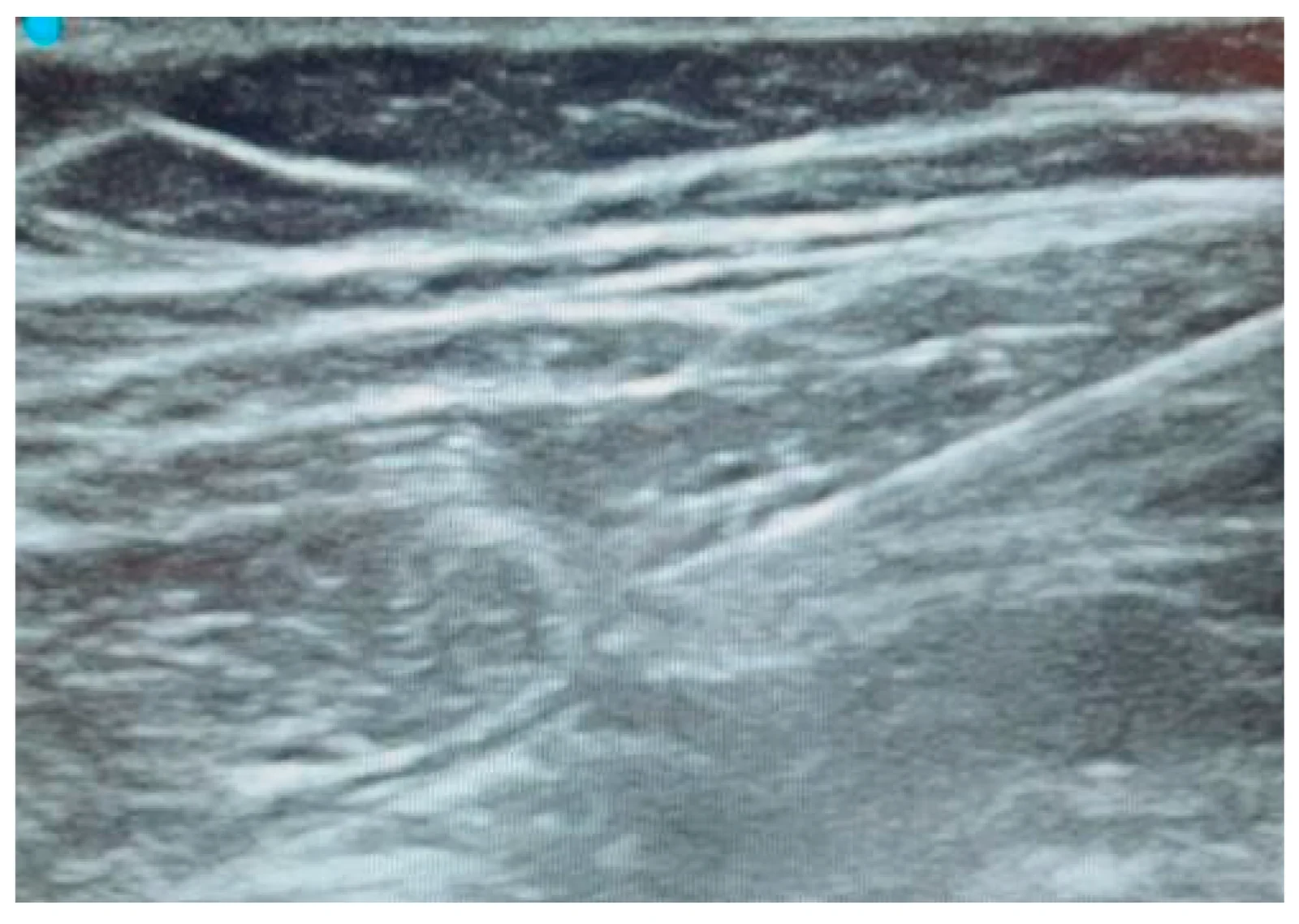
Entrance of spinal needle.

**Figure 5:**
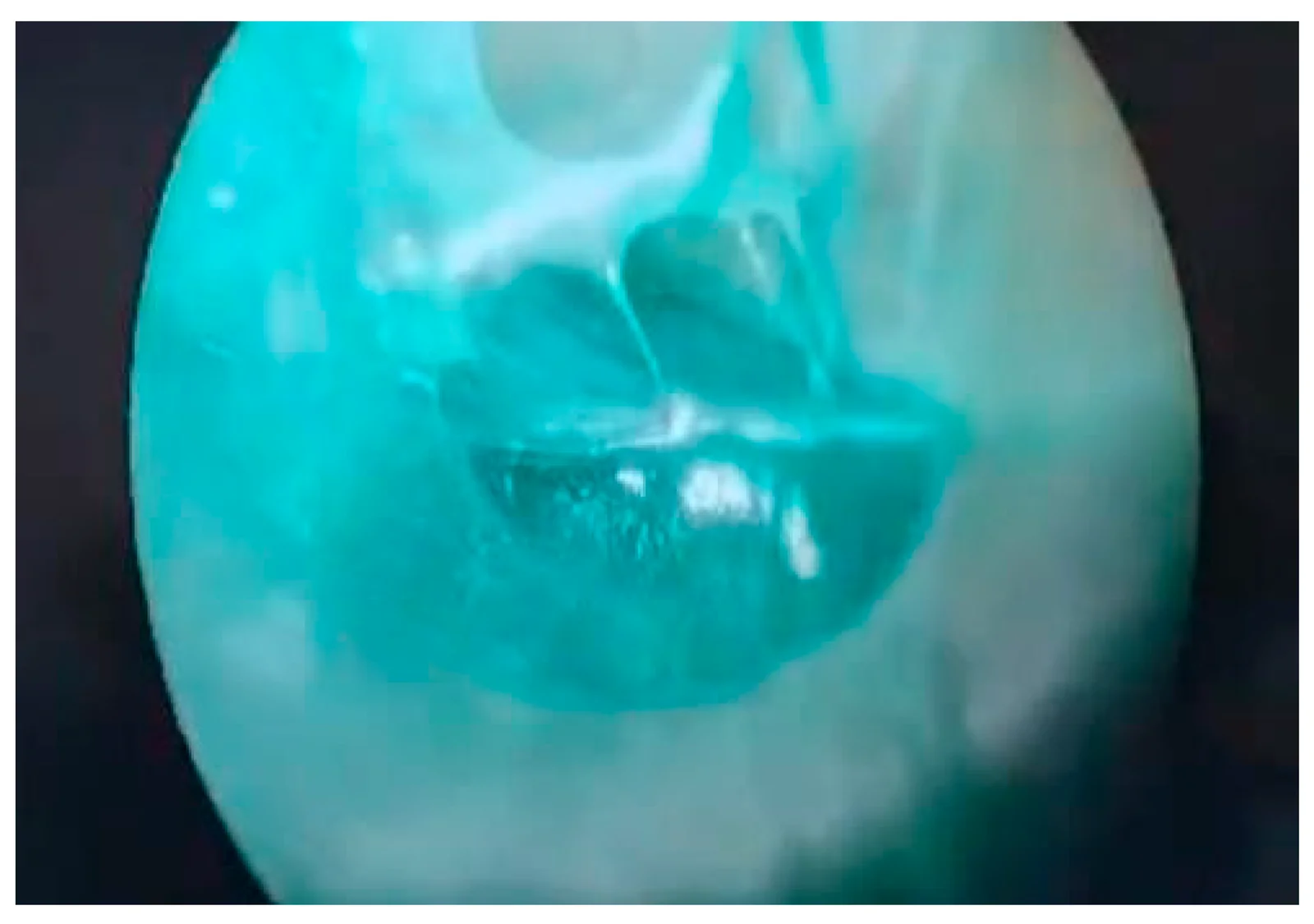
Injection of indocyanine green (with sterile water).

Lumbar sympathectomy is currently regarded as a surgical option for the management of plantar hyperhidrosis refractory to medical therapy. Among minimally invasive approaches, the most commonly adopted retroperitoneal technique relies on the use of a balloon dissector to create the working space after the placement of the first trocar, followed by carbon dioxide insufflation and insertion of additional trocars for the dissection of the lumbar sympathetic chain [7]. A combined intraperitoneal–retroperitoneal approach has also been described, in which the first trocar is placed under intraperitoneal visualization before proceeding with retroperitoneoscopic dissection [7]. In an effort to improve procedural safety and anatomical orientation during the access phase, we developed a modified retroperitoneoscopic technique. All procedures were performed under general anesthesia with the patient in the supine position. The surgeon and assistant stood on the operative side, while the monitor was positioned on the contralateral side. A sensor was placed on the ipsilateral foot to allow intraoperative monitoring of plantar temperature and perfusion (Figure 1). Two 5 mm operative trocars were used. Initial access was obtained under direct vision using an optical trocar [8]. As part of the preoperative anesthesiology-assisted protocol, an initial retroperitoneal space was created under ultrasound guidance. A spinal needle was advanced into the retroperitoneum, and 15 mL of a solution containing 25 mg of indocyanine green diluted at 1:20 with sterile water was injected, followed by 60 mL of air (Figure 2 and Figure 3). Particular attention was paid to avoiding inadvertent vascular injection (Figure 4). This maneuver facilitated entry of the optical trocar into the retroperitoneal space and was intended to reduce the risk of iatrogenic injury during initial access (Figure 5). Carbon dioxide insufflation was then established at low pressure (12 mmHg), and the remaining trocar was inserted under direct intracavitary vision. The intervention starts trying to reach the psoas plane with blunt shots of the optic to minimize blunt trauma on the peritoneum.

The psoas major muscle served as the principal retroperitoneal anatomical landmark. Dissection was carried out along the anterior surface of the psoas fascia, with anterior mobilization of the peritoneum, extraperitoneal fat, and ureter. The psoas muscle is treated as a reference until reaching its tendon implants on the vertebrae where the lumbar sympathetic chain can be identified by careful blunt dissection, medial to the psoas major muscle, sometimes beneath para-aortic or paracaval lymphatic tissue. Once the sympathetic trunk had been identified, it was isolated cranially to the superior border of the third lumbar vertebra and caudally to the inferior border of the fifth lumbar vertebra. The target lumbar sympathetic trunk was then temporarily clamped using a laparoscopic atraumatic clamp in order to assess the immediate physiologic response and confirm the adequacy of the target level. The effect of temporary clamping was evaluated through changes in plantar temperature and perfusion, which were typically observed within approximately 60 s. The perfusion index is measured using a special pulse oximeter placed on the big toe, while the temperature is measured with an infrared thermometer. Both values are then compared to the corresponding ones on the opposite foot (perfusion on the other big toe and temperature on the same area of the sole). When these changes were considered consistent with effective sympathetic interruption, a definitive titanium clip was applied to the sympathetic trunk. Procedures involving the clamping of the lumbar sympathetic trunk for hyperhidrosis have been performed at our facility for years; the technique itself remains unchanged, but we now use this initial ultrasound guidance to make the procedure easier and safer, while also allowing us to use one less trocar.

## 3. Results

**Figure 6:**
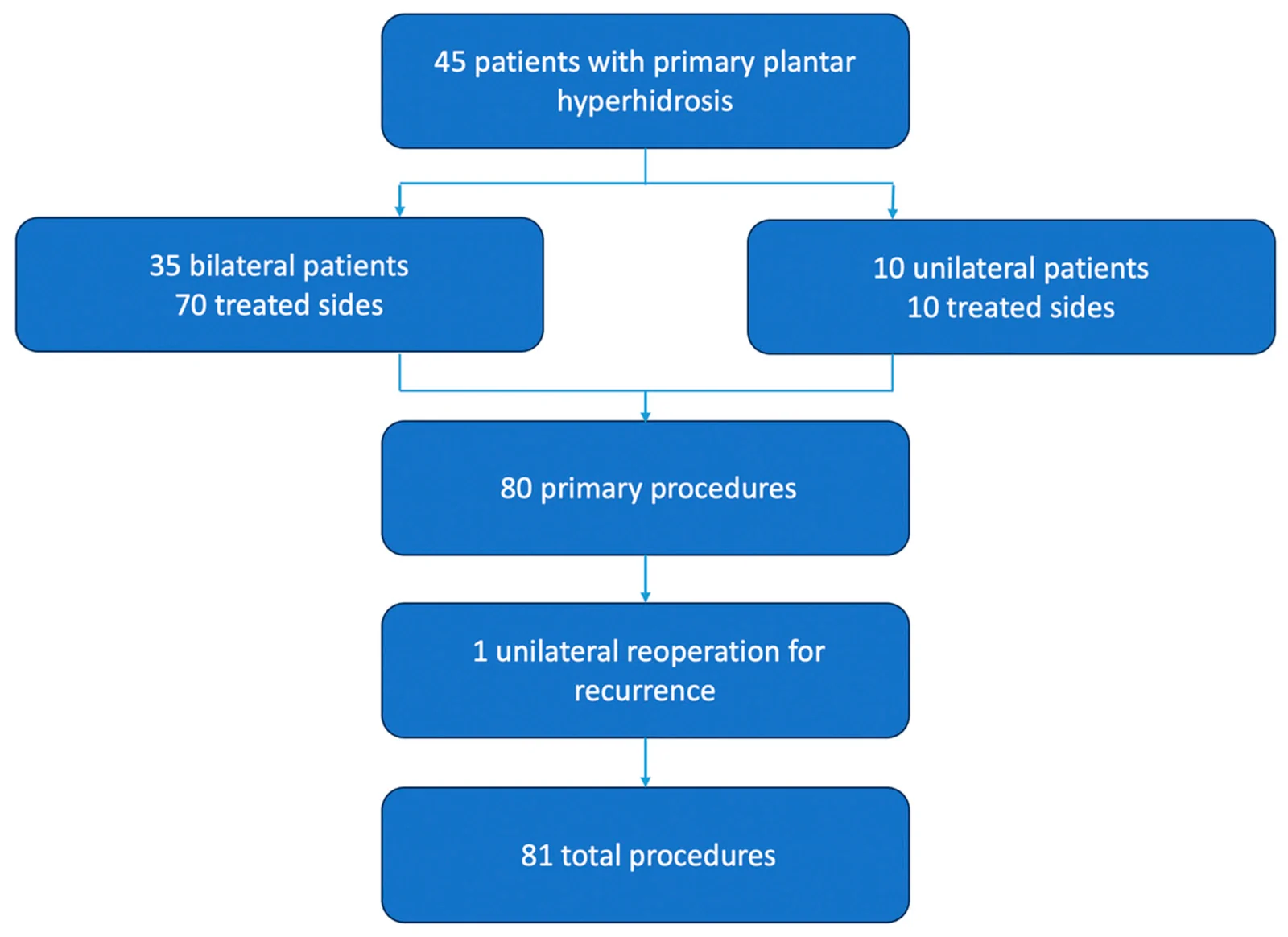
Study cohort and procedural flow. Forty-five patients underwent Lumbar Sympathetic Chain Clamping for primary plantar hyperhidrosis. Thirty-five patients received bilateral treatment and 10 received unilateral treatment, accounting for 80 primary treated sides. One additional unilateral procedure was subsequently performed for recurrent symptoms, resulting in 81 procedures overall.

Between June 2019 and May 2024, a total of 45 patients underwent lumbar sympathectomy for primary plantar hyperhidrosis using the described technique. Patient- and procedure-level characteristics are summarized in Table 1 and Figure 6. Overall, 81 procedures were performed, including 35 bilateral procedures (70 sides), 10 unilateral procedures, and one unilateral reoperation for recurrent symptoms. Perioperative outcomes are reported in Table 2. All patients resumed oral intake and autonomous ambulation on the day of surgery and were discharged within 24 h according to institutional protocol. No intraoperative or postoperative complications were observed. Specifically, no cases of hemorrhage, organ injury, infection, or need for surgical revision were recorded. The patients were regularly followed up over the following months (1 or more), and all of them reported improvement and resolution of their foot sweating.

## 4. Discussion and Limitations

Lumbar sympathectomy remains a valuable surgical option for selected patients with plantar hyperhidrosis refractory to conservative treatment, although its adoption is less widespread and its technique less standardized than thoracoscopic sympathectomy [3,6]. In this study, we describe a modified retroperitoneoscopic approach combining ultrasound-guided retroperitoneal access, indocyanine green-assisted space creation, and temporary selective clamping of the lumbar sympathetic trunk before definitive clipping. The minimally invasive nature of the procedure is significant regarding the number of trocars used; typically, three trocars are employed (including a 10 mm optical trocar), whereas, thanks to these improvements, we are able to use only two 5 mm trocars. In our preliminary single-center experience, the technique was associated with favorable perioperative outcomes, with no complications and discharge within 24 h in all patients. Its main advantages lie in the access phase and in intraoperative confirmation of the target level, improving upon traditional retroperitoneoscopic techniques based on balloon dissection or combined intraperitoneal–retroperitoneoscopic approaches [7]. Our modification was developed with the aim of making the initial access more controlled and reproducible by using ultrasound-guided needle placement, instillation of diluted indocyanine green and air, and optical trocar insertion under direct vision [8]. In our view, these technical modifications are intended to make retroperitoneoscopic lumbar sympathectomy safer and less invasive by facilitating retroperitoneal access, improving anatomical orientation, reducing the need for traumatic dissection and additional trocars, and allowing intraoperative confirmation of the target level before definitive interruption. Temporary atraumatic clamping provides immediate physiologic feedback through changes in plantar temperature and perfusion, helping confirm the effectiveness of the selected target. Although perfusion index monitoring appears promising, its use is not yet standardized and warrants further investigation. Postoperative recovery was rapid, with all patients resuming oral intake and ambulation on the day of surgery and being discharged within 24 h. These findings support the feasibility of this minimally invasive approach, which aims to maximize procedural safety while minimizing surgical trauma and ensuring intraoperative verification of treatment efficacy [3,6]. This study has several limitations, including its single-center design and the absence of a comparison group. Nevertheless, it provides a practical refinement of the retroperitoneoscopic approach to lumbar sympathectomy for plantar hyperhidrosis, focusing on improved retroperitoneal access, anatomical orientation, and intraoperative confirmation of the target level. These preliminary results support further prospective comparative studies to assess the reproducibility, long-term effectiveness, and broader applicability of the technique.

## 5. Conclusions

In this preliminary single-center experience, modified retroperitoneoscopic lumbar sympathectomy based on ultrasound-guided retroperitoneal access, indocyanine green-assisted creation of the working space, and temporary selective clamping of the lumbar sympathetic trunk proved to be a feasible and safe technique for the surgical treatment of primary plantar hyperhidrosis. These technical refinements may facilitate retroperitoneal entry, improve anatomical orientation, and provide immediate functional confirmation of the target sympathetic level before definitive clipping. Future prospective multicenter studies with standardized symptom evaluation, validated patient-reported outcome measures, and longer follow-up are warranted to confirm the reproducibility, durability, and comparative clinical value of this approach.

## Figures and Tables

**Table 1:** Patient and procedural characteristics.

Characteristic	Value
Patients, *n*	45
Age, years, mean (range)	35 (19–62)
Male sex, *n* (%)	29 (64.4)
Female sex, *n* (%)	16 (35.6)
Patients undergoing bilateral treatment, *n* (%)	35 (77.8)
Patients undergoing unilateral treatment, *n* (%)	10 (22.2)
Primary-treated sides, *n*	80
Reoperations for recurrent symptoms, *n*	1
Total procedures, *n*	81

Data are presented as number (%), unless otherwise indicated. Thirty-five patients underwent bilateral treatment, accounting for 70 treated sides, whereas 10 underwent unilateral treatment. One additional unilateral procedure was performed for recurrent symptoms.

**Table 2:** Perioperative and clinical outcomes.

Outcome	Value
Resumption of oral intake on the day of surgery, *n* (%)	45 (100)
Autonomous ambulation on the day of surgery, *n* (%)	45 (100)
Discharge within 24 h, *n* (%)	45 (100)
Intraoperative complications, *n* (%)	0 (0)
Postoperative complications, *n* (%)	0 (0)
Hemorrhage, *n* (%)	0 (0)
Organ injury, *n* (%)	0 (0)
Postoperative infection, *n* (%)	0 (0)
Surgical revision for complications, *n* (%)	0 (0)
Reoperation for recurrent symptoms, *n* (%)	1 (2.2)

## Data Availability

The original contributions presented in this study are included in the article. Further inquiries can be directed to the corresponding author.
